# Chiral Dielectric Metasurfaces for Highly Integrated, Broadband Circularly Polarized Antenna

**DOI:** 10.3390/s21062071

**Published:** 2021-03-16

**Authors:** Bruno Ferreira-Gomes, Osvaldo N. Oliveira, Jorge Ricardo Mejía-Salazar

**Affiliations:** 1 National Institute of Telecommunications (Inatel), Santa Rita do Sapucaí 37540-000, MG, Brazil; bruno.gomes@mtel.inatel.br; 2 São Carlos Institute of Physics, University of São Paulo, P.O. Box 369, São Carlos 13560-970, Brazil; chu@ifsc.usp.br

**Keywords:** chirality, dielectric resonator antennas, metasurfaces

## Abstract

We report on the design of a low-profile integrated millimeter-wave antenna for efficient and broadband circularly polarized electromagnetic radiation. The designed antenna comprises a chiral dielectric metasurface built with a 2×2 arrangement of dielectric cylinders with slanted-slots at the center. A broadbeam high-gain with wide axial ratio (AR)<3 dB bandwidth was reached by pairing the electric and magnetic resonances of the dielectric cylinders and the slanted slots when excited by an elliptically polarized driven-patch antenna. This electric-magnetic pairing can be tuned by varying the cylinders diameter and the tilting and rotation angles of the slanted slots. The simulation results indicate impedance-matching bandwidths up to 22.6% (25.3–31.6 GHz) with 3-dB AR bandwidths of 11.6% (26.9–30.2 GHz), which in terms of compactness ($0.95\lambda_{0}\times 0.95\lambda_{0}$) and performance are superior to previous antenna designs. Since the simulations were performed by assuming materials and geometries easily implementable experimentally, it is hoped that circularly polarized antennas based on chiral metasurfaces can be integrated into 5G and satellite communications.

## 1. Introduction

The quest for antennas that are cost-effective, compact, and efficient for a broad bandwidth with a specific radiation pattern relies on the ability to manage millimeter-wave (mm-wave) electromagnetic field interactions. To transfer mm-wave antennas from research laboratories to the market of mobile infrastructures (for 5G communication) and satellite communication, it is crucial to address polarization mismatches and suppress multipath interferences. This may in principle be reached with circularly polarized (CP) antennas working at different ranges in the GHz regime [1,2,3,4,5,6,7], but two major limitations must be addressed. The first is to reach high-quality CP radiation with high gain within a broad bandwidth (BW). The quality of a CP electromagnetic wave is defined in terms of its axial-ratio (AR), i.e., the ratio between the major and minor axes of the polarization ellipse. The closer AR is to unity (0 dB) the higher the CP quality, and in practice electromagnetic fields have been considered to be CP for AR below 3 dB [1,2,3,4,5,6,7]. As for the second drawback, one has to reduce the size and Ohmic losses (inherent in metallic inclusions) of mm-wave antennas to produce them in high-throughput, integrable platforms. Dielectric resonator antennas (DRAs) made with high-permittivity ($\varepsilon ∼10\cdots 10^{2}$) materials (e.g., ceramics) can concentrate and radiate CP electromagnetic fields with small losses [8]. However, the integration of these CP-DRAs into modern wireless systems is hampered by the need for multiple resonators and a multi-feed mechanism to achieve wide bandwidths [9,10,11,12,13]. Another possibility to address these limitations is to employ chiral systems in the design of CP antennas [14,15]. Chirality refers to the handedness of an object which cannot be superimposed with its mirror image as in left- (LCP) and right-handed circularly polarized (RCP) light. The interaction of chiral electromagnetic fields with a chiral scatterer depends on this handedness. This may be used to simultaneously improve the handedness of an electromagnetic source while enhancing its electromagnetic field intensity, which can be reached through the proper engineering of suitable chiral structures [16]. In fact, chirality concepts have been used to control electromagnetic wave propagation in the microwave and optical domains [17,18,19], which serves as inspiration for CP antenna design.

In this communication, we demonstrate theoretically a new concept to produce integrated, highly efficient mm-wave CP antennas in which we employ metasurfaces [20,21]. These types of surfaces were proven useful for several applications, including gain enhancement [22] and phase rectifying [23]. In contrast to previous antenna designs using bulky metallic-based chiral metasurfaces [14,15], we exploit the pairing of electric and magnetic resonances of high-permittivity (ε=16) cylindrical resonators with slanted slots at the center. An analogous concept in nanophotonics yielded an enhanced broadband near- and far-field chiroptical activity [18]. To reach optimized radiation patterns with high gain and wide AR-BW, we used a 2×2 arrangement of slotted cylinders. Through numerical calculations we show that this concept can be readily applied in antenna arrays by using a metal cavity surrounding each unit cell. The results for the system with metal cavity show promising improvements in gain and AR amplitudes and bandwidths. For comparison purposes, simulations were made using two commercial software packages for antenna design, namely CST Studio and ANSYS HFSS, and the results were in excellent agreement. CST Studio uses a time domain (transient) solver based on the finite integration technique (FIT) with a hexahedral mesh, while HFSS employs the finite element method (FEM) with tetrahedral mesh elements. As we shall demonstrate, antennas made with a chiral dielectric metasurface (CDM) are promising for broadband and efficient CP mm-wave radiation.

## 2. Antenna Design

### 2.1. Configuration

**Figure 1:**
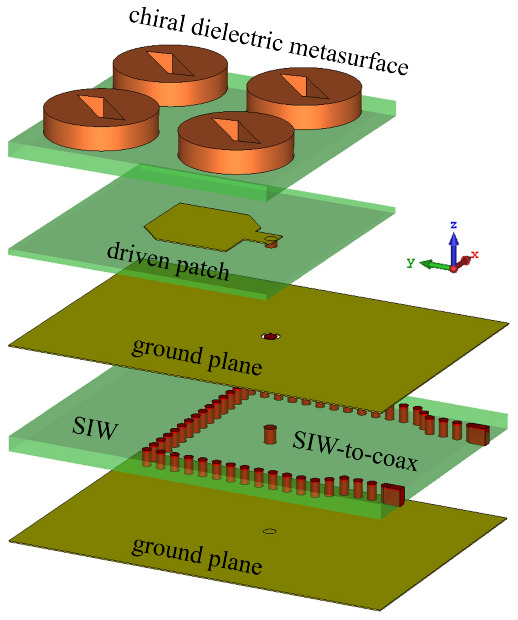
3D geometric view of the proposed antenna element fed by a SIW ideal connector.

Figure 1 shows a zoomed view of the antenna designed to have the center frequency at 28.5 GHz to enable high-throughput applications in 5G and satellite communications.

**Figure 2:**
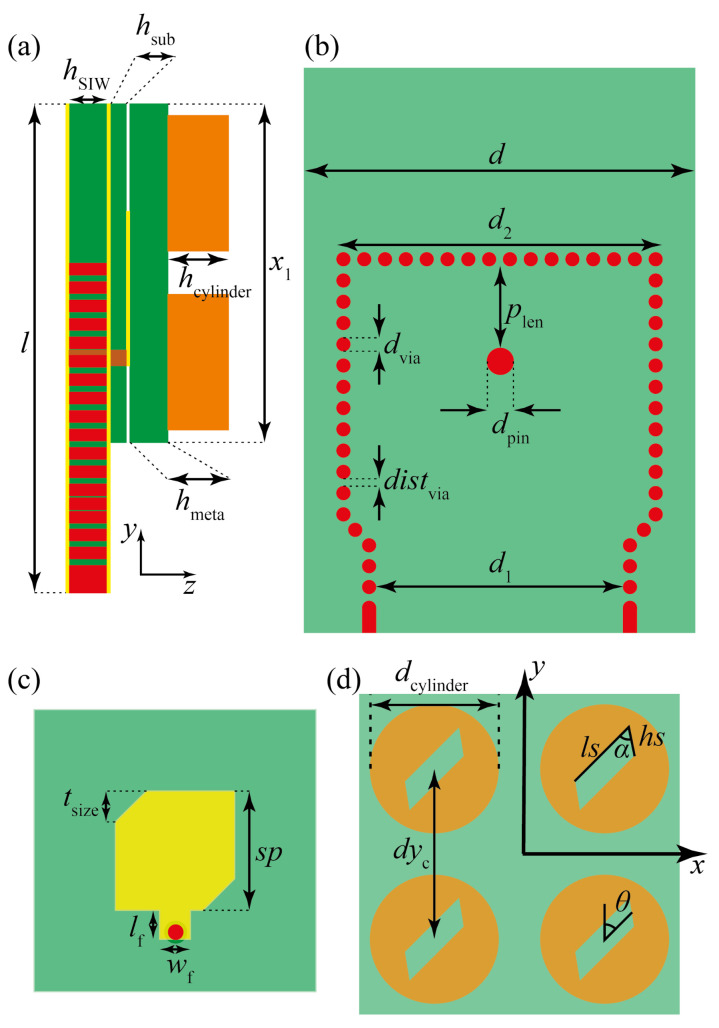
(**a**) Parametric side view of the antenna layers divided as: (**b**) SIW-to-coax feeding, (**c**) driven patch layer and (**d**) chiral dielectric metasurface layer. The design parameters are $x_{1}=10$ mm, d=10 mm, $d_{2}=7.5$ mm, $p_{\mathrm{len}}=2.15$ mm, $d_{\mathrm{via}}=0.3$ mm, $d_{\mathrm{pin}}=0.4$ mm, $dist_{\mathrm{via}}=0.19$ mm, $d_{1}=5.71$ mm, $t_{\mathrm{size}}=0.86$ mm, sp=3.3 mm, $l_{f}=0.85$ mm, $w_{f}=0.8$ mm, ls=2.1 mm, hs=0.8 mm, $\alpha =60^{\circ }$ and $\theta =45^{\circ }$.

The antenna consists of five parts referred to as (i) the bottom ground plane; (ii) a substrate integrated waveguide (SIW) with a SIW-to-coax feeding; (iii) a ground plane film; (iv) a coax-fed driven patch antenna; and (v) a chiral dielectric metasurface consisting of a 2×2 array of slotted dielectric cylinders. The ground plane films were considered to be made of lossy copper. A top-view and side-view of the main building elements of the antenna design are presented in Figure 2.

The SIW-based feeding layer is etched on a slab of Rogers RT/duroid 5880 ($\epsilon_{r}=2.2$, tanδ=0.0009), with thickness $h_{\mathrm{SIW}}=0.5$ mm. A ground plane, $h_{\mathrm{sub}}=0.035$ mm thick, was used to separate the SIW-feeding and the coax-fed driven-patch antenna. A CDM is stacked at the top of the structure, consisting of four slotted cylinders made of a ceramic dielectric material with $\epsilon_{r}=16$, layered on a low-permittivity substrate of RT/duroid 5880 ($h_{\mathrm{meta}}=0.5$ mm). The cylinders had a height $h_{\mathrm{cylinder}}=0.8$ mm and diameter $d_{\mathrm{cylinder}}=3.6$ mm, and were separated by a center-to-center distance of $dy_{c}=4.8$ mm. The slots at the center of the cylinders were parallelograms whose sides were labeled as ls and hs and the tilting angle is α. The slots are rotated by an angle θ with respect to the *y*-axis, as depicted. CDM is fed by a driven patch localized below the low-permittivity layer at the center of the metasurface. The patch made of lossy copper had rectangular sides sp and corner cuts $t_{\mathrm{size}}$. The coax-to-patch feeding line has a width $w_{f}=0.8$ mm and length $l_{f}=0.85$ mm. The SIW-feeding structure is amenable to integration with planar front-end circuits. The SIW structure was designed as a rectangular waveguide input, forming a SIW-to-coax transition where the distance between the pin and the shorting wall was tuned for impedance matching [24,25]. The dimensions of the SIW-feeding and the remaining parameters are listed in the caption of Figure 2. It is worth mentioning that with the antenna size taken as $0.95\lambda_{0}\times 0.95\lambda_{0}$ ($\lambda_{0}$ at the center frequency of 28.5 GHz), our concept enables the application of phase-arrayed antenna systems by designing the structure to be directly fed by a coax-wire.

### 2.2. Working Principle and Enhancement of ARBW

Recent approaches for enhancement of AR bandwidth in metallic antennas have employed two pair slots, as explained in Reference [21]. Alternatively, dielectric resonators with high-permittivity are known to support electric and magnetic resonances of different orders [8,10], in analogy with cylindrical nanostructures [18]. The near-field overlapping between nearby cylinders resembles the electronic-bands from well-localized atomic orbitals in the tight-binding model [26,27] in electromagnetic platforms known as metasurfaces. Here, we use dielectric cylinders with a slanted slot at the center to produce integrable, highly efficient and broad ARBW CP antennas. An analogous concept was exploited in nanophotonic platforms to produce giant enhancement of chiroptical effects [18]. The concept is based on the coupling of the electric and magnetic modes of the resonators (considered to be dipoles in a qualitative approximation) which can be expressed as

(1) $$\begin{array}{c} \tilde{p}=\tilde{\alpha }\tilde{E}-i\tilde{G}\tilde{B},\;\;\;\;\;\;\;\tilde{m}=\tilde{\chi }\tilde{B}+i\tilde{G}\tilde{E}, \end{array}$$

where $\tilde{p}$ and $\tilde{m}$ are the electric and magnetic dipoles, respectively. $\tilde{\alpha }$ and $\tilde{\chi }$ are the complex electric polarizability and magnetic susceptibility, while $\tilde{G}$ corresponds to the mixed electric-magnetic dipole polarizability. $\tilde{E}$ and $\tilde{B}$ are the complex electric and magnetic fields. The radiated electromagnetic energy has, therefore, a term ∼G˜″ImE˜·B˜, where ${\tilde{G}}^{\prime \prime }$ stands for the imaginary part of $\tilde{G}$. It is just at the slanted sides hs where $\tilde{E}$ and $\tilde{B}$ become mixed [18], allowing the tuning of $\tilde{G}$ through the tilting angle α (see Figure 2d). The electromagnetic fields exciting the CDM are produced by a single narrow-band driven-patch antenna with truncated corners, thus being constantly fed by two orthogonal linearly polarized modes (AR≳5), as will be shown later.

## 3. Results and Discussion

**Figure 3:**
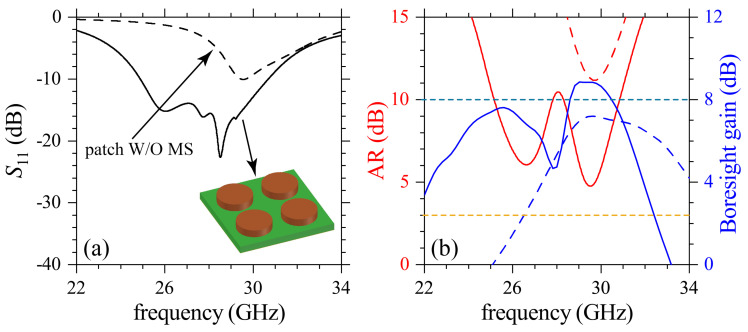
Numerical results for (**a**) $S_{11}$ and (**b**) AR and boresight gain. Solid and dashed lines are for the patch antenna with and without (W/O) the DR metasurface. Simulations were carried out using the commercial software CST Studio. Horizontal dashed lines in (**b**) are eye guides for AR=3 dB and boresight gain = 8 dB.

The individual contributions from the building components in the antenna design can be studied by analyzing the results in Figure 3 for $S_{11}$, AR and boresight gain for the patch without (W/O) metasurface (dashed lines) and with the metasurface of solid cylinders (solid lines) depicted in the inset of Figure 3a. Calculations in this figure were made using the commercial software CST Studio. Results for $S_{11}$ are presented in Figure 3a, whereas the corresponding AR and boresight-gain are shown in Figure 3b. Poor values for the impedance-matching ($S_{11}≳-10$) and gain (<8) are observed for the patch W/O metasurface. The antenna with the metasurface containing the solid cylinders improves the impedance-matching, AR values and boresight gains, in addition to an improved bandwidth performance. Such improvements are due to the dielectric cylindrical resonators and their corresponding near-field interactions. However, the radiation and AR performance are still low in all of these results, and additional strategies are required to design useful CP antennas.

**Figure 4:**
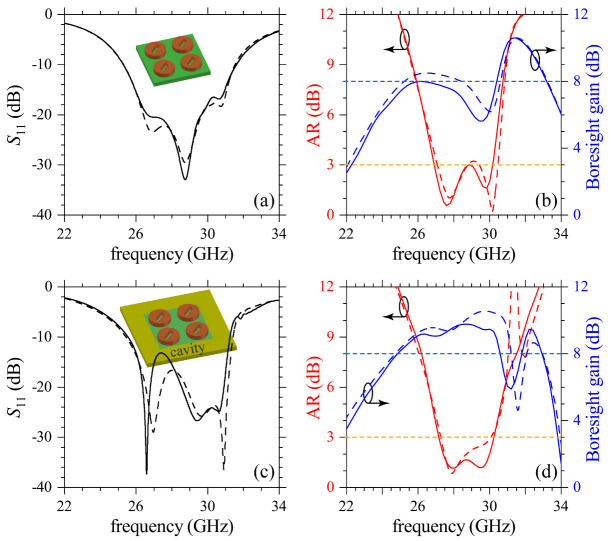
Numerical results for $S_{11}$, AR and boresight gain are presented for the CDM-based antenna (**a**,**b**) with and (**c**,**d**) without the metal cavity. Simulations from CST Studio and ANSYS HFSS are indicated by solid and dashed lines. Horizontal dashed lines in (**b**,**d**) are eye guides for AR=3 dB and boresight gain = 8 dB.

**Figure 5:**
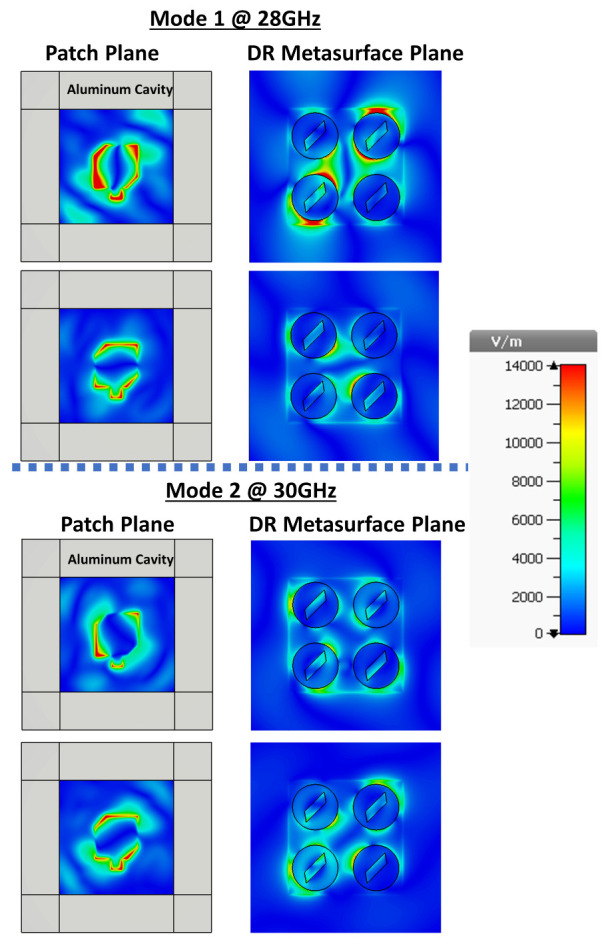
Simulated **E**-field of the antenna element at orthogonal phases for both CP modes and both planes: Patch and DR Metasurface Planes.

Following the reasoning of chiral dielectric metasurfaces for enhanced chiroptical effects [18], we considered slanted slots etched at the center of each cylinder, as schematized in the inset of Figure 4a. This mechanism not only improved AR but also the whole operating performance of the antenna, as it can be seen from Figure 4a,b. Calculations from CST Studio and ANSYS HFSS are presented with solid and dashed lines, respectively. Negligible differences are observed due to different convergences of the software packages. The applicability of this CDM-based CP antenna is demonstrated in Figure 4c,d with AR and gain corresponding to the system surrounded by a metal cavity (considered to be made of Al). This cavity surrounding the four-element CDM enables integration into antenna arrays by suppressing the mutual coupling between adjacent elementary cells. Although a small decrease was induced by the cavity in the impedance $\mathrm{BW}=\left[25.5\;\mathrm{GHz},31.3\;\mathrm{GHz}\right]$ and 3-dB $\mathrm{ARBW}=\left[27.1\;\mathrm{GHz},30.3\;\mathrm{GHz}\right]$, in relation to the impedance $\mathrm{BW}=\left[25.3\;\mathrm{GHz},31.6\;\mathrm{GHz}\right]$ and 3-dB $\mathrm{ARBW}=\left[26.9\;\mathrm{GHz},30.2\;\mathrm{GHz}\right]$ for the system without cavity, the corresponding gain (≳9) and AR (AR≪3) were considerably improved. Moreover, a gain notch from Figure 4b was shifted out of the bandwidth range of interest when using the cavity, which certainly improves the CP radiation performance. Figure 5 shows the E-field distribution for the two orthogonal modes of the patch coupled to the CDM. These fields were calculated for the frequencies at which AR achieves the best results, according to Figure 4d. For illustrative purposes, results are presented for E-field distribution at the patch and CDM planes, and with radiation of an LCP electromagnetic field. As it can be inferred, an RCP antenna can be developed using the mirror image of CDM and the patch antenna. The evanescent behavior of the electromagnetic field in the cavity is also shown.

**Figure 6:**
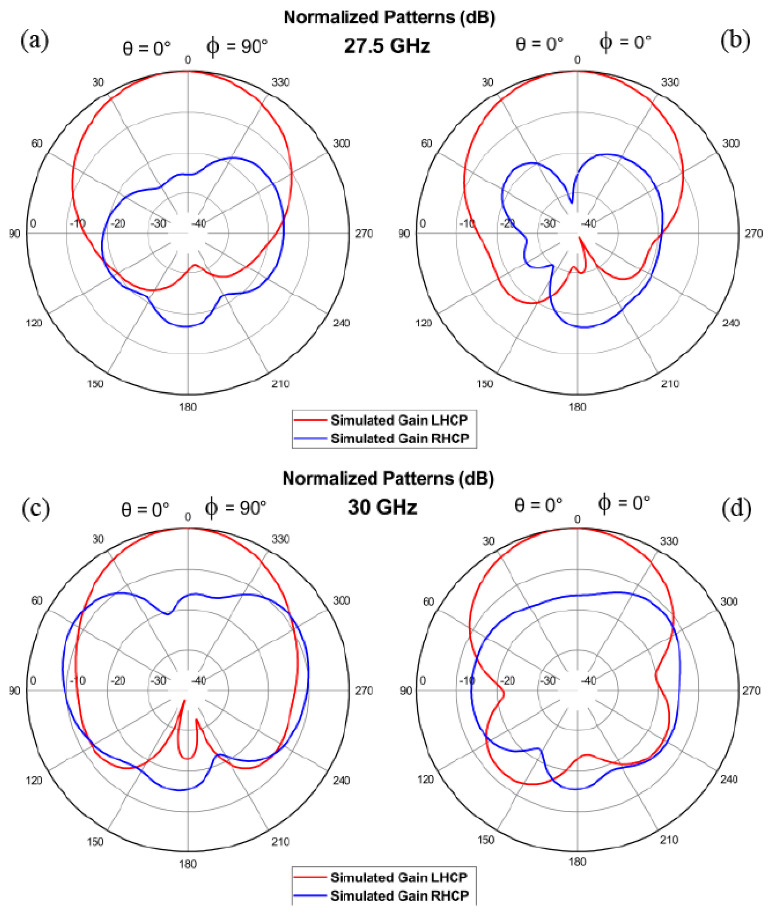
Simulated normalized radiation patterns at 27.5 GHz (**a**,**b**) and 30 GHz (**c**,**d**) for both cut-planes.

The numerical results from hereon were obtained with the commercial software CST Studio for the antenna without the cavity. Results of the LCP (co-polarized) and RCP (cross-polarized) radiation patterns for the CDM-based antenna are presented for 27.5 GHz and 30 GHz in Figure 6a–d, respectively. For the co-polarized radiation at the $\phi =90^{\circ }$-plane, a 3-dB power beamwidth of $66.3^{\circ }$ (in the range from $38.3^{\circ }$ to $-28^{\circ }$) is observed for 27.5 GHz (Figure 6a) while for 30 GHz it is $58.7^{\circ }$ (from $31^{\circ }$ to $-27.7^{\circ }$) (Figure 6c). The condition AR≤3-dB is met when the difference between the co-polarized and cross-polarized patterns is ≥15-dB. Therefore, there is a high AR performance in the whole 3-dB power beamwidth for the 27.5 GHz mode, but not for 30 GHz which is limited to $43^{\circ }$ (from $23^{\circ }$ to $-20^{\circ }$). In the case of $\phi =0^{\circ }$-plane, there is nearly the same 3-dB power beamwidth for both modes, $66.3^{\circ }$ (in the range from $36.3^{\circ }$ to $-30^{\circ }$) for 27.5 GHz and $62.7^{\circ }$ (in the range from $36^{\circ }$ to $-26.7^{\circ }$) for 30 GHz. However, the 3-dB AR performance for 30 GHz exhibits a very narrow beamwidth of $36^{\circ }$ (from $24^{\circ }$ to $-12^{\circ }$), in contrast to 27.5 GHz that stays with at least 15-dB difference between the co-polarized and cross-polarized radiation patterns in the whole 3-dB power beamwidth. Hence, the area covered by the 27.5 GHz mode is larger than for the 30 GHz.

## 4. Parametric Study of the Proposed Design

**Figure 7:**
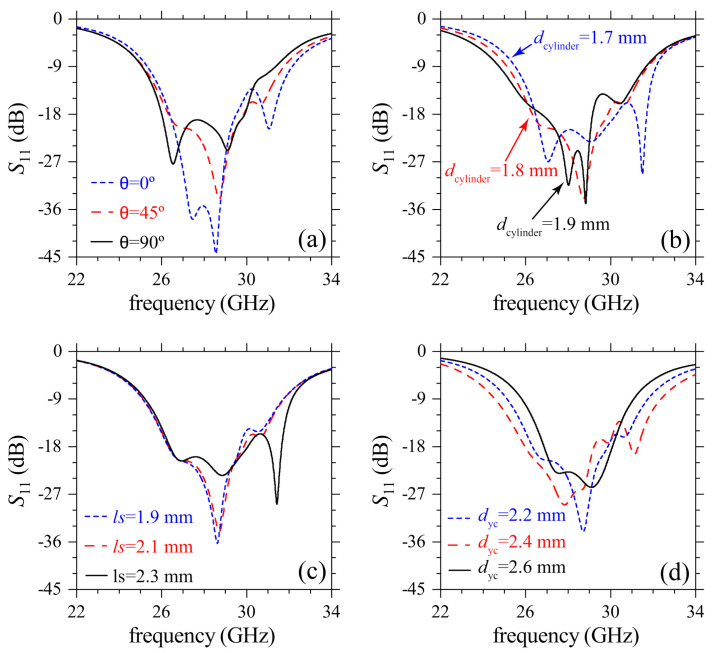
Simulated parametric study for the reflection coefficient of the proposed antenna. (**a**) variation of θ, (**b**) variation of $d_{cylinder}$, (**c**) variation of ls and (**d**) variation of $dy_{c}$.

**Figure 8:**
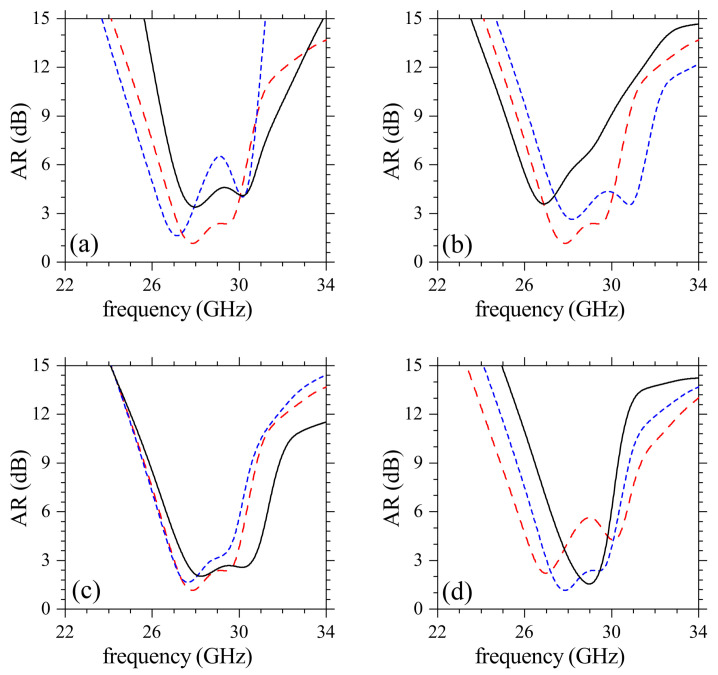
Simulated parametric study for AR of the proposed antenna, in relation to the structure in Figure 7. The variation of (**a**) θ, (**b**) $d_{cylinder}$, (**c**) ls and (**d**) $dy_{c}$ was the same as in Figure 7a–d, respectively.

To understand how different design parameters affect the functioning of the meta-antenna, we plotted $S_{11}$ and AR for different values of θ, $d_{\mathrm{cylinder}}$, *l*s and $dy_{c}$ in Figure 7 and Figure 8. There is a slight narrowing of the impedance bandwidth for increasing θ in Figure 7a, in contrast with the loss of CP radiation (AR>3) in Figure 8a. This dependence of AR on θ highlights the importance of this parameter when designing the metasurface to obtain high-performance CP radiation. Upon increasing $d_{\mathrm{cylinder}}$ the modes in $S_{11}$ are discretized with loss in the ability to generate CP radiation as indicated by the poor AR values (AR>3) in Figure 8b. The latter behavior is explained by recalling that the CDM modes should be matched with the two orthogonal modes from the driven patch for a proper coupling and field re-radiation. Another important parameter in the design is the length ls of the slots, which can be varied to tune AR (and its bandwidth) to its optimum values with small changes of $S_{11}$, as noted in Figure 7c and Figure 8c. The interaction of nearby cylinders in CDM depends on $dy_{c}$ which then affects the quality of CP radiation. Figure 7d and Figure 8d point to a decrease in the ability to produce CP mm-waves upon increasing $dy_{c}$. Excitation of several out-of-phase resonances occurs for very small $dy_{c}$ which despite widening the impedance bandwidth, negatively affect AR.

The competitiveness of our CDM-based antenna is demonstrated by comparing with recent proposals for CP antennas in Table 1, where results are given for the central frequency, impedance bandwidth (BW), ARBW, 2-dB gain bandwidth, and peak gain. We avoid comparing sizes because of the nature of the antenna and the corresponding operating frequencies. However, we emphasize that the CDM in this work has a reduced size while maintaining its efficiency. Moreover, there is a high peak gain with low gain variation in a wide bandwidth which is broader than in reports in the literature. Therefore, we expect that this work will stimulate the use of CDMs to produce CP radiation in other antenna designs.

## 5. Conclusions

The introduction of a chiral metasurface on the design of low-profile CP DRAs was shown to yield a wide AR bandwidth, high-gain and low AR values. Of special relevance is the compactness of the CP antenna designed, suitable for mobile communication systems. The concepts and design were validated through numerical simulations using two commercial software packages, and no significant difference was noted in the results obtained with CST Studio and ANSYS HFSS. From these simulations one learns that the CP antennas can be tunable and integrated into front-end circuits or phase-arrayed systems (directly fed by a coax-wire). The design also included materials and conditions which are readily available for implementation, and therefore we may expect the fabrication of CP antennas for high throughput 5G and satellite communications in the Ka-band.

## Figures and Tables

**Table 1:** Comparison of performance with other CP Antennas.

Designs	Freq.	BW	3dB-ARBW	2dB-Gain-BW	Peak Gain
	(GHz)	(%)	(%)	(%)	(dBic)
Ref. [5]	3.5	21	8.5	19	6.5
Ref. [7]	26	12.5	26	18	7.5
Ref. [11]	5.5	25.4	22.8	34.2	7.7
Ref. [12]	11.7	27.8	24	16.5	10
this work *	28.5	22.6	11.6	34	8.5
this work **	28.5	20.4	11.2	19.4	10.4

*—W/O cavity, **—with cavity.
